# microRNA-874 suppresses tumor proliferation and metastasis in hepatocellular carcinoma by targeting the DOR/EGFR/ERK pathway

**DOI:** 10.1038/s41419-017-0131-3

**Published:** 2018-01-26

**Authors:** Yi Zhang, Yangchao Wei, Xuan Li, Xingsi Liang, Liming Wang, Jun Song, Xiuzhong Zhang, Chong Zhang, Jian Niu, Pengbo Zhang, Zeqiang Ren, Bo Tang

**Affiliations:** 1 Department of General Surgery, Affiliated Hospital of Xuzhou Medical University, Xuzhou, 221000 China; 2 Department of Hepatobiliary Surgery, Affiliated Hospital, Guilin Medical University, Guilin, 541001 China; 3 Department of Hepatobiliary Surgery, Second Affiliated Hospital of Dalian Medical University, Dalian, 541000 China

## Abstract

The δ opioid receptor (DOR) is involved in the regulation of malignant transformation and tumor progression of hepatocellular carcinoma (HCC). However, regulation of the DOR in HCC remains poorly defined. We found that miR-874 was identified as a negative regulator of the DOR, which is a direct and functional target of miR-874 via its 3′ untranslated region (UTR). Moreover, miR-874 was downregulated in HCC and its expression was inversely correlated with DOR expression. Downregulation of miR-874 was also associated with larger tumor size, more vascular invasion, a poor TNM stage, poor tumor differentiation, and inferior patient outcomes. Functionally, overexpression of miR-874 in the HCC cell line SK-hep-1 inhibited cell growth, migration, in vitro invasion, and in vivo tumorigenicity. Furthermore, miR-874 overexpression suppressed the DOR, resulting in a downregulated epidermal growth factor receptor (EGFR) and extracellular signal-regulated kinase (ERK) phosphorylation. The EGFR activator—epidermal growth factor (EGF)—can rescue the proliferation and migration suppression induced by miR-874 overexpression, and the rescue effects of the EGF were blocked by an ERK inhibitor. Our study results suggest that miRNA-874 is a negative regulator of the DOR that can suppress tumor proliferation and metastasis in HCC by targeting the DOR/EGFR/ERK pathway, which may be a potential target for HCC treatment.

## Introduction

Hepatocellular carcinoma (HCC) is the sixth most prevalent cancer and the third most frequent cause of cancer-related death^1^. Most of these deaths are caused by the progression of the tumor to metastatic disease. Thus, understanding the molecular mechanisms underlying cancer progression and metastasis may contribute to the discovery of more effective intervention methods and liver cancer treatment targets.

The delta-opioid receptor (DOR) is a type of G protein-coupled receptor (GPCR), and the human DOR gene is located on chromosome 6q24-25. The coding region of DOR is 1119 bp, which encodes 372 amino acid residues^2^. In addition to its distribution throughout the human body, DOR is also present in various human cancers and involved in malignant transformation or tumor progression. The activation of DOR is reported to increase the survival rate of the neuroblastoma–glioma hybrid cell line through activation of the RTK/PI3K/Akt signaling pathway^3^. The activation of opioid receptors (delta and mu opioid receptors) by morphine increases cell proliferation and invasion of non-small-cell lung cancer by increasing phosphorylation of the epidermal growth factor receptor (EGFR)^4^. Moreover, the DOR was found to be highly expressed in human breast cancer, and activating the DOR promoted its proliferation by increasing phosphorylation levels of extracellular signal-regulated kinases (ERKs)^5^.

Our group has performed many studies on the function of the DOR in HCC. We have demonstrated that the DOR is extensively expressed in human HCC cells and that its functional status directly affects the proliferation, apoptosis, invasion, and migration of these cells^6^. Moreover, DOR-mediated inhibition of apoptosis was accessed through the ERK pathway^7^.

microRNAs (miRNAs) are endogenous non-coding small RNA molecules that regulate the expression of target genes. This regulation typically occurs via imperfect base-pairing to the 3′ untranslated region (UTR) of a target mRNA, which leads to mRNA degradation or the inhibition of translation^8^. Studies have demonstrated that miRNAs regulate the expression of many target genes and are involved in the initiation, progression, and metastasis formation of various cancers^9^. However, there are no reports of miRNAs targeting the DOR. In the present study, we used miRNA target-prediction algorithms to identify miR-874, which controls DOR expression through binding to the 3′-UTR of the DOR in HCC. We also investigated the contribution of miR-874 to the proliferation and metastasis of HCC and the underlying mechanism.

## Results

### Identification of miRNAs regulating DOR expression in HCC cell lines

**Fig. 1:**
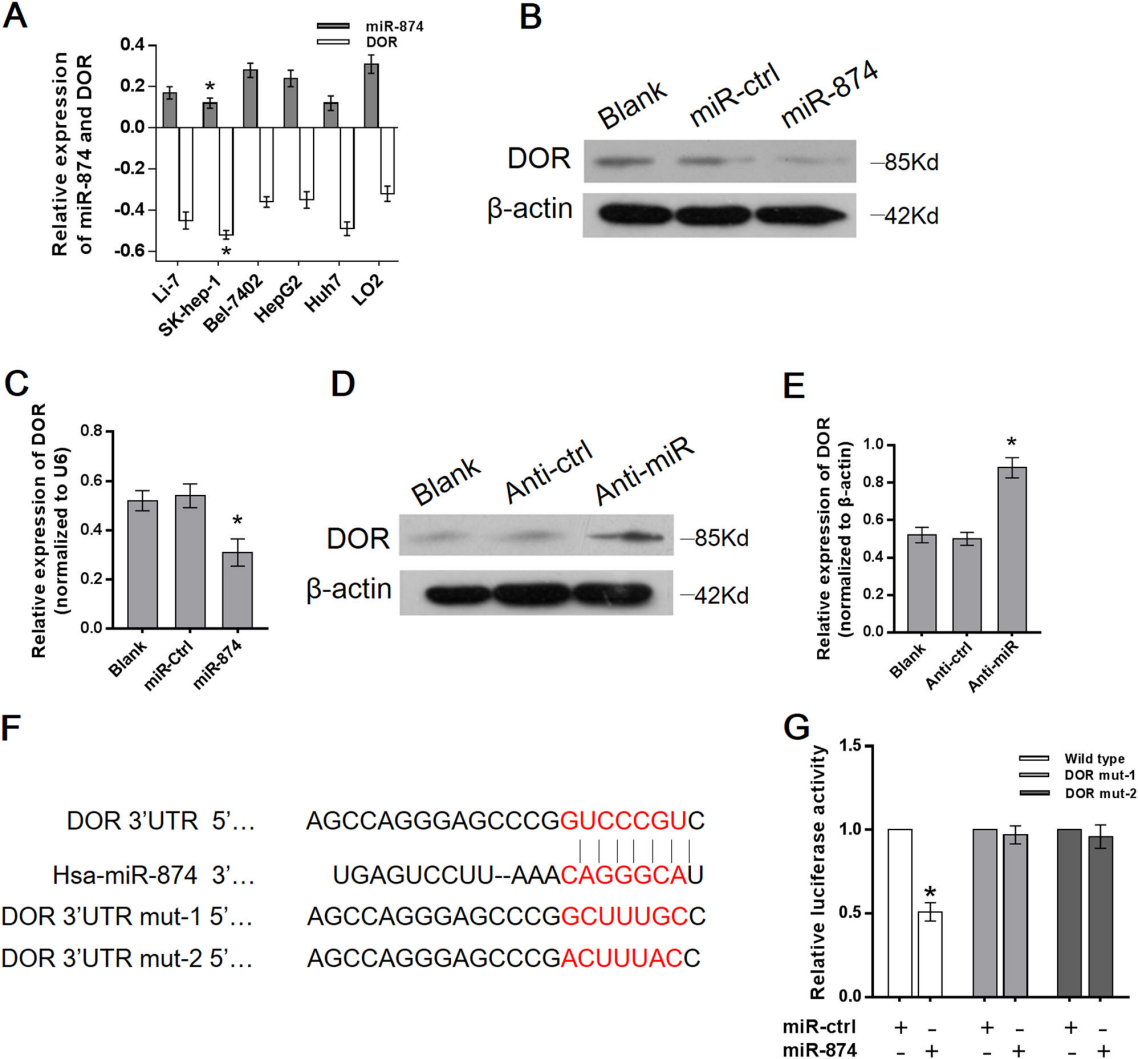
miR-874 downregulated DOR expression by directly targeting its 3′-UTR **a** Expression of miR-874 and the DOR in HCC cell lines were detected by qRT-PCR in six cell lines. miR-874 and DOR expression were normalized to U6 and β-actin, respectively. **b**, **c** The protein and mRNA levels of the DOR decreased after transfection with the miR-874 mimic, as demonstrated by Western blot and qRT-PCR. **d**, **e** The protein and mRNA level of the DOR increased after transfection with the miR-874 inhibitor, as demonstrated by Western blot and qRT-PCR. **f** The predicted sites of miR-874 binding to the 3′-UTR of the DOR were detected using bioinformatics prediction tools. The mutated site in the 3′-UTR of the DOR is shown. **g** The effect of miR-874 on luciferase activity induced by the pMIR-DOR-wt, pMIR-DOR-mut-1, and pMIR-DOR-mut-2 reporter plasmids in SK-hep-1 cells was measured via luciferase reporter gene assays. Data are shown as the mean ± SD of three replicates (*p < 0.05)

To identify miRNAs that may regulate DOR expression in HCC, we performed an online database search (microRNA.org and TargetScan 6.2) and identified two miRNAs (miR-874 and miR-184) targeting the 3′-UTR of the DOR. Among these, miR-874 was proven to negatively correlate with the DOR in these six cell lines (Bel-7402, SK-hep-1, HepG2, Huh7, Li-7, LO2). Of these cell lines, SK-hep-1 had the lowest miR-874 expression and highest DOR expression (Fig. 1a). We used SK-hep-1 to perform the following experiments. DOR expression was clearly reduced when miR-874 expression was restored with miR-874 mimic transfection (Fig. 1b, c), but expression increased when miR-874 was knocked down with miR-874 inhibitor transfection (Fig. 1d, e). In contrast, miR-184 showed no correlation with the DOR (data not shown). These results suggested that miR-874 specifically decreased DOR expression.

### miR-874 downregulated DOR expression by directly targeting its 3′-UTR

To gain insight into the mechanism by which miR-874 inhibits the DOR, we identified the miR-874 binding site at position 323–329 of the DOR 3′-UTR (Fig. 1f). The target region sequence of the DOR 3′-UTR (wild-type) or mutated sequence 1 (DOR mut-1) or 2 (DOR mut-2) was cloned into a luciferase reporter vector. These constructed reporter vectors were co-transfected with the miR-874 mimic or miR-ctrl into the SK-hep-1 cell line. The overexpression of miR-874 resulted in a significant decrease in the luciferase activity of the construct containing wild-type 3′-UTR of the DOR. This regulation was abolished when the nucleotides in the putative binding site were mutated (Fig. 1g), indicating that the miR-874-mediated regulation of DOR expression depended on its binding to a specific seed region in the DOR 3′-UTR.

### miR-874 was downregulated in HCC tissues and correlated with survival

**Fig. 2:**
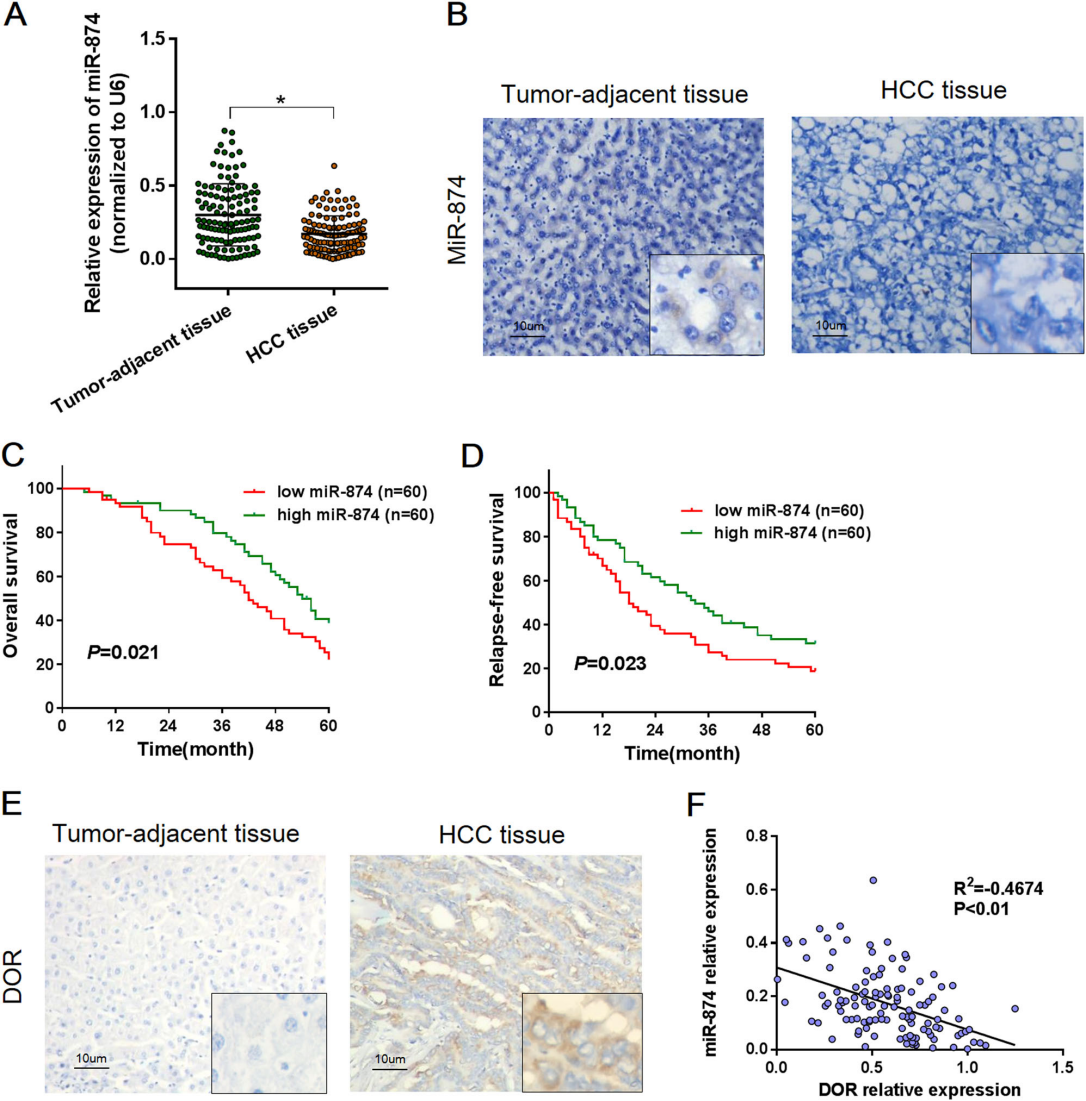
miRNA-874 expression was inversely associated with DOR expression in HCC tissues, and high miR-874 expression predicted longer RFS and OS **a** The relative expression levels of miR-874 were assessed via qRT-PCR in 120 paired HCC and tumor-adjacent tissues. **b** Analysis of miR-874 expression in HCC tissues and tumor-adjacent tissues by ISH. **c**, **d** Kaplan–Meier analysis revealed five-year OS rates of 22.05 and 38.70% and five-year RFS rates of 11.77 and 31.34% in the low and high miR-874 expression groups, respectively, suggesting that the OS and RFS rates of the low miR-874 expression group were higher than those of the high miR-874 expression group (*p* < 0.01). **e** Analysis of DOR expression in HCC tissues and tumor-adjacent tissues by IH. The DOR was overexpressed in cytoplasm of colon cancer tissues but nearly absent from paired tumor-adjacent tissues. **f** Expression of miR-874 was inversely correlated with the mRNA level of the DOR (*r* = −0.467, *p* < 0.01, Spearman’s correlation analysis). Data are shown as the mean ± SD of three replicates (*p < 0.05)

We examined the expression of miR-874 in HCC tissues, and mature miR-874 was detected in 120 paired HCC and tumor-adjacent tissues using quantitative real time RT-PCR (qRT-PCR) and in situ hybridization (ISH). The qRT-PCR results showed that expression of miR-874 was significantly downregulated in cancer tissue compared to the adjacent tissue (*p* < 0.01, Fig. 2a). Furthermore, ISH analysis confirmed the expression pattern of miR-874 in tissues (Fig. 2b), indicating that downregulation of miR-874 is a frequent event in HCC tissues.

We further analyzed the clinicopathological significance of miR-874 in HCC tissues. The relationship between miR-874 expression levels and the clinicopathological characteristics of HCC patients are summarized in Table 1. The patients were stratified into 2 groups based on the median miR-874 expression levels. The miR-874 levels were negatively associated with tumor size (*p* = 0.043), tumor stage (*p* = 0.001), tumor differentiation (*p* = 0.037), and vascular invasion (*p* = 0.046).

**Table 1:** Relationship between miRNA-874 and clinicopathological parameters in 120 HCC patients

Variables	All cases	miR-874 expression		*χ* ^2^	p*
		High *N* = 60	Low *N* = 60		
*Age(years)*					
≥50	20	11	9	0.784	0.624
<50	100	49	51		
*Gender*					
Male	80	36	44	0.023	0.121
Female	40	24	16		
*Tumor number*					
Single	101	54	47	0.010	0.080
Multiple	19	6	13		
*Etiology*					
viral	94	46	48	0.902	0.658
Non-viral	26	14	12		
*Serum AFP (ng/ml)*					
≤200	66	29	37	0.032	0.142
>200	54	31	23		
*Tumor stage*					
I/II	69	43	26	0.000	0.001
III/IV	51	17	34		
*Tumor size (cm)*					
≤5	53	32	21	0.003	0.043
>5	67	28	39		
*Tumor differentiation*					
Well	48	30	18	0.075	0.037
Moderate	35	17	18		
Poor	35	13	24		
*Vascular invasion*					
Yes	36	13	23	0.003	0.046
No	84	47	37		

*P probability, from *χ*^2^ test.

We conducted a five-year follow-up with patients. Kaplan–Meier analysis revealed that the five-year Overall Survival (OS) rates were 45.27 and 70.80% (Fig. 2c), and that the five-year Relapse-free Survival (RFS) rates were 44.96 and 64.13% (Fig. 2d) in the low and high miR-874 expression groups, respectively. This finding suggests that the OS and RFS rates of the low miR-874 expression group were higher than those of the high miR-874 expression group (*p* < 0.05). We also evaluated the prognostic significance of the DOR in HCC patients. The patients were stratified into high and low groups based on the median DOR mRNA expression; the results revealed that patients with high DOR expression had shorter OS and RFS, in a trend opposite that of miR-506 (Fig. S2A).

### Clinical correlation of miR-874 and DOR expression

We also examined DOR expression in 120 paired HCC and tumor-adjacent tissues via immunohistochemistry (IHC). High DOR expression (95/120) was detected in the cytoplasm of malignant cells, whereas low DOR expression (84/120) was observed in cells in the para-carcinoma tissue (Fig. 2e). We further investigated the relationship between miR-874 expression and DOR expression. We found that the expression of miR-874 was inversely correlated with DOR expression (Fig. 2f), suggesting that miR-874 negatively regulates DOR expression.

### miR-874 inhibited proliferation, migration, and invasion of HCC cells

Next, we explored the potential role of miR-874 in regulating HCC progression. We detected invasion ability in six cell lines, and the results confirmed that the SK-hep-1 cell line exhibits higher invasive capacity than the other five cell lines based on a transwell assay (*p* < 0.05, Fig. S1A). Spearman’s correlations suggested that miR-874 was negatively correlated with the invasiveness of these six cell lines and that DOR expression was positively correlated (Fig. S1B, C). The results show that the SK-hep-1 cell line is more aggressive than other cell lines, and we used it to perform the following experiments.

**Fig. 3:**
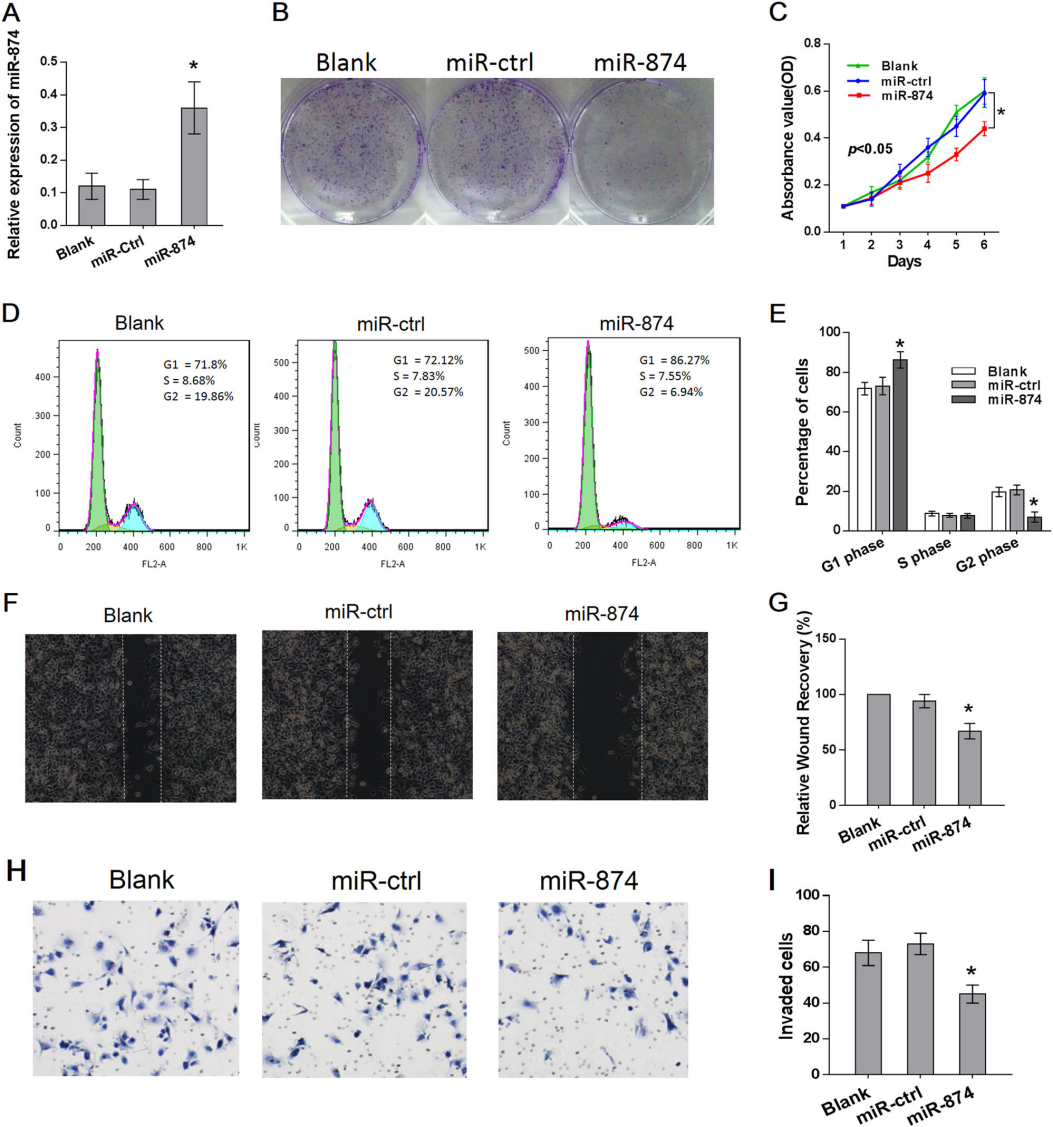
Ectopic expression of miR-874 inhibited proliferation, migration, and invasion of HCC **a** The level of miR-874 was detected via qRT-PCR after transfection of a miR-874 mimic for 48 h. **b** miR-874 overexpression suppressed in vitro colony formation of SK-hep-1 cells. **c** MTT assay showed that transfection of a miR-874 mimic suppressed proliferation of SK-hep-1 cells. **d**, **e** The effect of miR-874 on cell-cycle distribution of SK-hep-1 cells was monitored via flow cytometry. The miR-874-overexpressing SK-hep-1 cells were arrested at the G1 phase of the cell cycle. **f**, **g** Wound-healing assays showed that migration of SK-hep-1 cells was significantly reduced after restoration of miR-874 expression. **h**, **i** Transwell assays showed that the invasion of SK-hep-1 cells was significantly reduced after restoration of miR-874 expression. Data are shown as the mean ± SD of three replicates (*p < 0.05)

The miR-874 mimic was transfected into SK-hep-1 cells, and the transfection efficiency was confirmed by qRT-PCR (Fig. 3a), while miR-874 overexpression resulted in significant tumor growth inhibition in SK-hep-1 cells without affecting the apoptotic rate (Figs. 3b, c; S2B). In contrast, SK-hep-1 cells transfected with a miR-874 inhibitor displayed increased growth (Fig. S1D). Cell-cycle distribution analysis showed that the miR-874-overexpressing SK-hep-1 cells were arrested at the G1 phase, with a corresponding reduction in the percentage of cells in the S and G2 phases (Fig. 3d, e).

In addition, the migration and invasion capacities were evaluated via wound-healing and transwell assays, respectively. The wound-healing assay demonstrated that the migration of miR-874-overexpressing SK-hep-1 cells in the wound was much slower than that of miR-ctrl-transfected and blank cells (Fig. 3f, g). The invasiveness of SK-hep-1 cells was assessed using a transwell assay and was significantly reduced after restoration of miR-874 expression (Fig. 3h, i). We also detected the expression of several EMT (epithelial to mesenchymal transition) markers by Western blot. The epithelial marker E-cadherin level increased and mesenchymal marker vimentin and N-cadherin decreased upon overexpression of miR-874 (Fig. S2D), suggesting that miR-874 inhibits EMT in SK-hep-1 cells.

### Restoration of DOR expression reversed proliferation and invasion of HCC cells

**Fig. 4:**
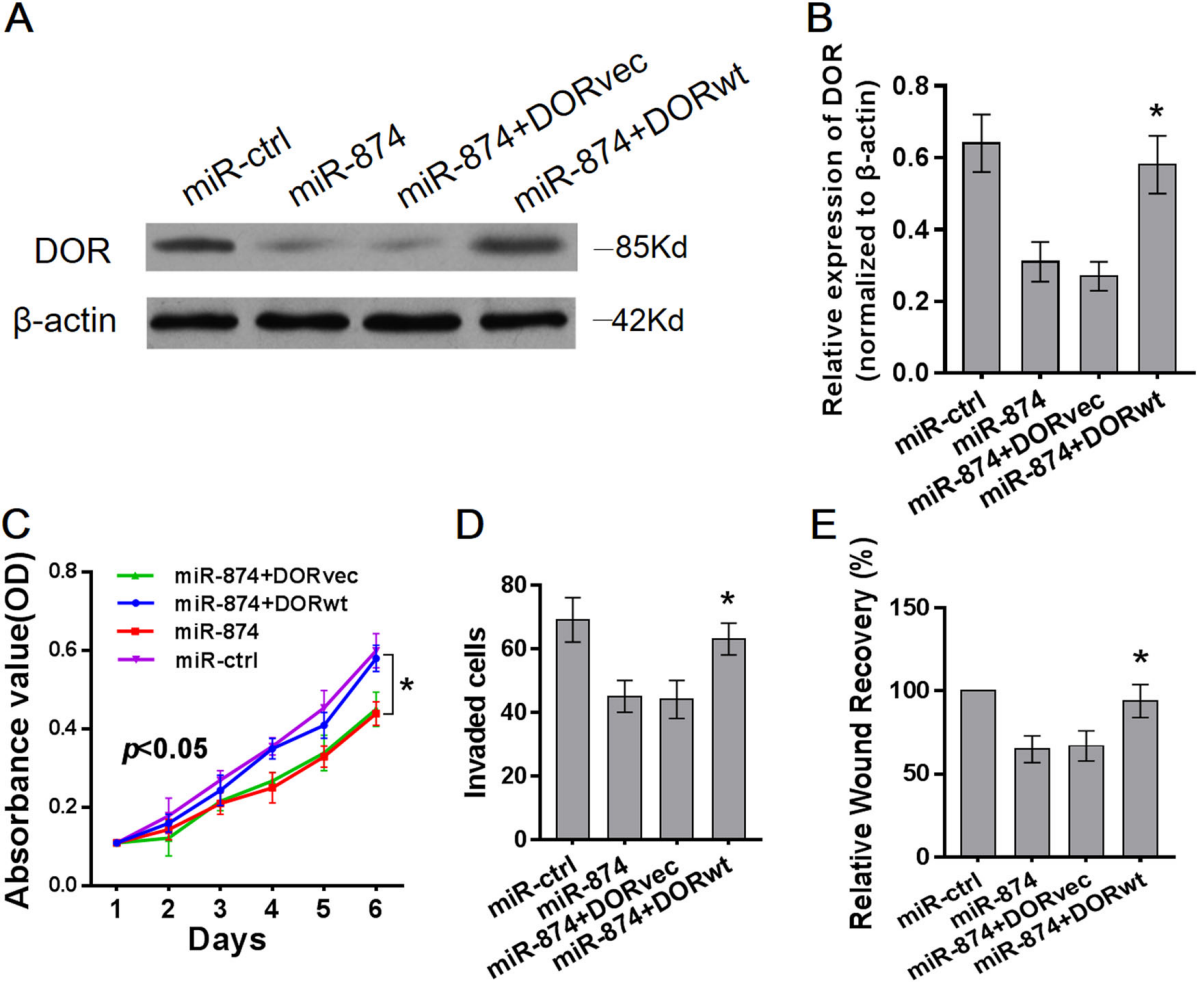
Restoration of DOR expression reversed proliferation and invasion of HCC cells **a**, **b** Protein and mRNA levels of the DOR increased after transfection with DOR plasmids into miR-874-overexpressing SK-hep-1 cells, as demonstrated by Western blot and qRT-PCR. **c** Proliferation of SK-hep-1 cells was reversed after transfection with DOR plasmids into miR-874-overexpressing SK-hep-1 cells, as demonstrated by MTT assay. **d** Transwell assay demonstrated that restoration of DOR expression ameliorated miR-874-induced suppression of SK-hep-1 cell invasion. **e** Wound-healing assay demonstrated that restoration of DOR expression ameliorated miR-874-induced suppression of SK-hep-1 cell migration. Data are shown as the mean ± SD of three replicates (*p < 0.05)

Our previous studies have reported that silencing DOR expression inhibited the proliferation and invasion of HCC cells^6^. We hypothesized that the phenotypes associated with miR-874 expression would be reversed by the restoration of DOR expression. Therefore, we constructed a DOR expression plasmid and co-transfected this plasmid into SK-hep-1 cells. DOR expression was confirmed by Western blot (Fig. 4a, b). The MTT, matrigel invasion, and wound-healing assays demonstrated that restoration of DOR expression significantly ameliorated the miR-874-induced suppression of SK-hep-1 cell proliferation, invasion, and migration, respectively (Fig. 4c–e). Therefore, the DOR has an important role in the proliferation and metastasis of SK-hep-1 cells, potentially by acting as a mediator of miR-874 function.

### miR-874 suppresses proliferation and migration via the DOR/EGFR/ERK pathway

We further elucidated the mechanism by which the miR-874-DOR axis regulates the proliferation and metastasis of HCC cancer. Our previous studies have shown that the DOR may regulate apoptosis through activation of the ERK signaling pathway^7^. Furthermore, it is reported that the DOR can induce phosphorylation of the EGFR in lung cancer, leading to downstream ERK phosphorylation^4^. Therefore, we speculated that the miR-874-DOR induced inhibition of proliferation and migration was associated with the EGFR–ERK pathway.

**Fig. 5:**
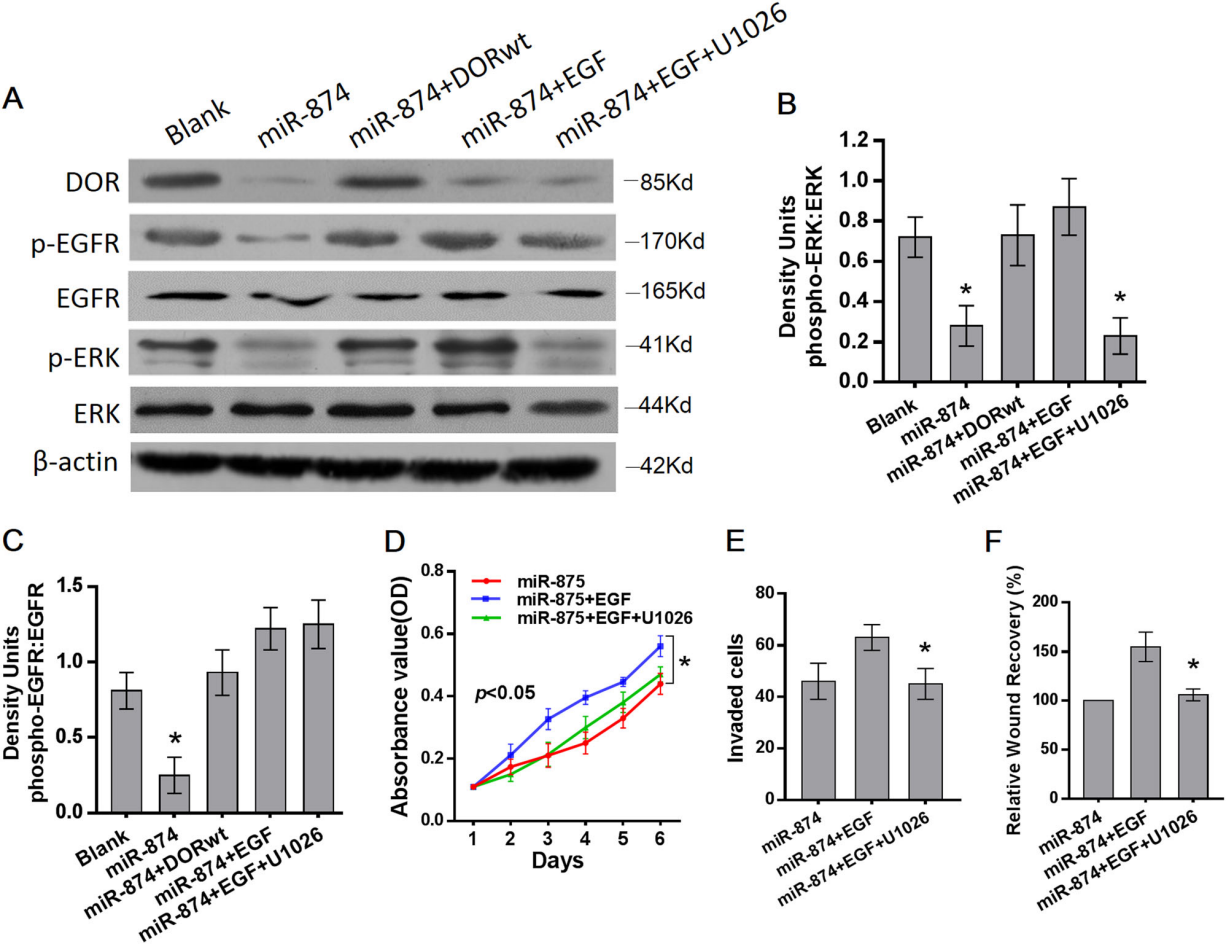
miR-874-DOR induced inhibition of proliferation and migration through EGFR–ERK pathway **a** Levels of the DOR, phosphorylated PKC, and phosphorylated ERK were detected using Western blot analysis. miR-874-overexpressed cells were treated with 20 ng/ml of EGF or combined with 10 µM of U1026. **b**,** c** Ratios of density units for each phospho- to total-protein are shown. Phosphorylation of the EGFR and ERK were downregulated by miR-874 overexpression, and ectopic DOR expression eliminated the promoting effect of miR-874 overexpression on phosphorylation level. The EGF significantly restored inhibition of phosphorylation of the EGFR and ERK induced by miR-101 overexpression. When miR-874-overexpressed SK-hep-1 cells were treated with EGF combined with U1026, EGF-induced phosphorylation of ERK was blocked without interfering with EGFR phosphorylation. **d** The proliferation-promoting effect of the EGF on miR-874-overexpressing SK-hep-1 cells was blocked by U1026, as demonstrated by an MTT assay. **e** The invasion-promoting effect of the EGF was blocked by U1026, as demonstrated by transwell assay. **f** The migration-promoting effect of the EGF was blocked by U1026, as demonstrated by wound-healing assay. Data are shown as the mean ± SD of three replicates (*p < 0.05)

Western blot showed that the phosphorylation of the EGFR and ERK was downregulated by miR-874 overexpression, and ectopic DOR expression eliminated the inhibiting effect of miR-874 overexpression on the phosphorylation level. An EGFR activator, the epidermal growth factor (EGF), significantly restored the inhibition of phosphorylation of the EGFR and ERK induced by miR-101 overexpression. When miR-874-overexpressed SK-hep-1 cells were pretreated with ERK inhibitor U1026 after the addition of the EGF, EGF-induced phosphorylation of ERK was blocked without interfering with EGFR phosphorylation (Fig. 5a–c). This finding suggests that miR-874 inhibits the DOR, which influences the EGFR–ERK pathway.

To demonstrate that the EGFR–ERK pathway is responsible for miR-874-triggered proliferation and migration inhibition, miR-874-overexpressing SK-hep-1 cells were pretreated with the EGF. MTT, transwell, and wound-healing assays demonstrated that the EGF restored the repression of proliferation and migration induced by miR-874 overexpression. When miR-874-overexpressing SK-hep-1 cells were pretreated with U1026 following the addition of the EGF, the restoring effect of the EGF was blocked by the ERK inhibitor (Fig. 5d–f), suggesting that miR-874 suppresses the proliferation and migration via the DOR/EGFR/ERK pathway.

### miR-874 inhibits tumorigenicity in vivo

**Fig. 6:**
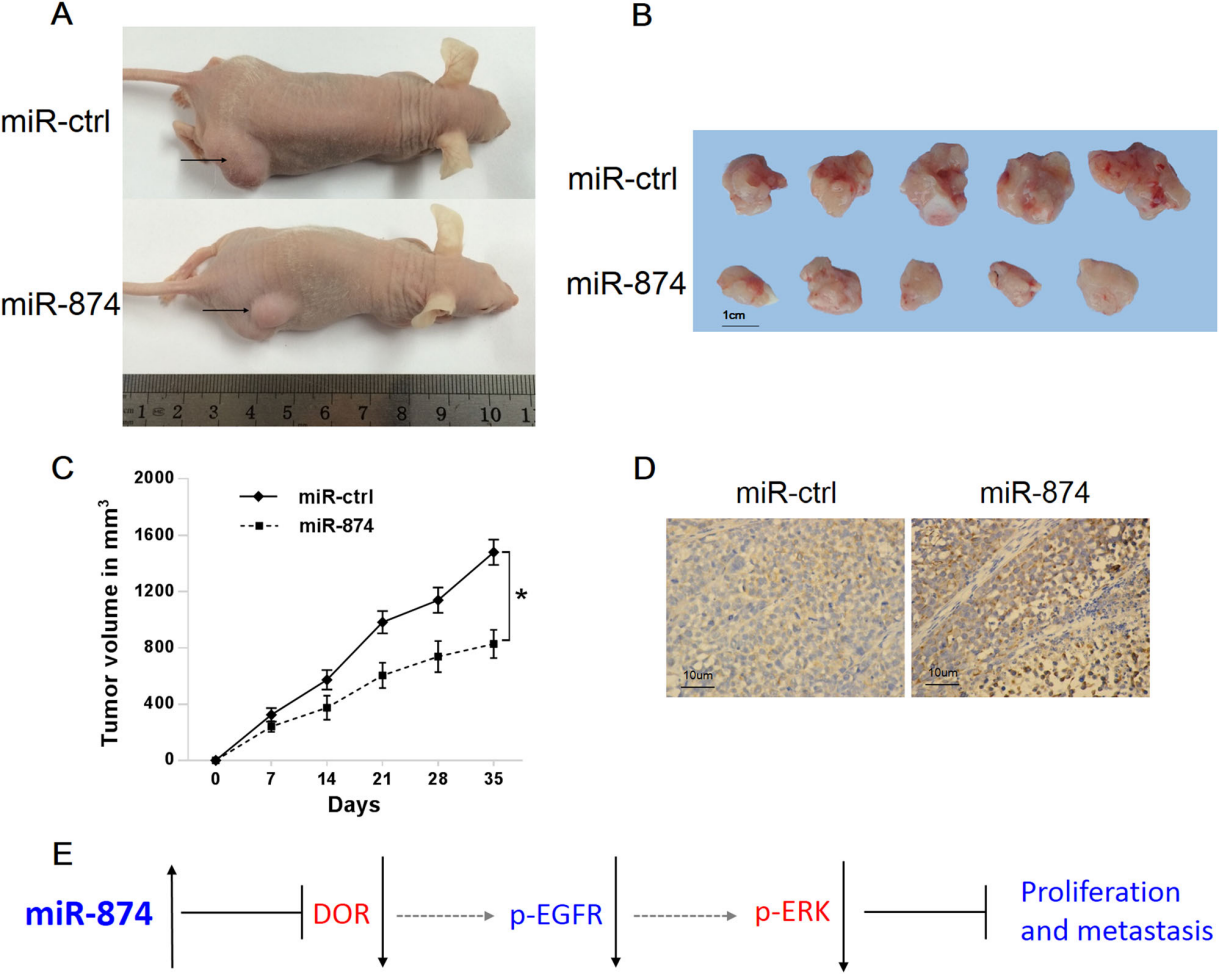
miR-874 inhibits tumorigenicity in vivo **a** Tumors formed in nude mice. SK-hep-1 cells stably overexpressing miR-874 or empty vector were injected into the flanks of nude mice, and the mice were killed after 5 weeks. **b** Surgically removed tumor tissues from nude mice 5 weeks post-inoculation. **c** Tumor volume at different time points after inoculation. **d** Histological images of resected tumors showed that tumor tissue consisted of HCC cells. **e** Postulated mechanism of miR-874 inhibiting proliferation and metastasis of HCC through DOR–EGFR–ERK pathways. Data are shown as the mean ± SD (*p < 0.05)

We then examined the role of miR-874 in tumorigenicity potential in vivo. SK-hep-1 cells stably overexpressing miR-874 were injected into the flanks of nude mice, and cells stably expressing an empty vector were used as a control. The mice were killed after 5 weeks, and the tumors were collected. The results showed that the mice injected with miR-874-overexpressing SK-hep-1 cells exhibited a significantly smaller tumor size than those injected with control cells (Fig. 6a–c). The histological images of the resected tumors showed that the tumor tissue consisted of HCC cells (Fig. 6d). Then, we sought to determine whether miR-874 inhibits tumor growth through the DOR/EGFR/ERK pathway. Immunohistochemical analysis of the DOR and Ki-67 showed higher expression in mice expressing an empty vector than in mice overexpressing miR-874 (Fig. S2C). Moreover, the phosphorylation of the EGFR and ERK were downregulated in the miR-874 group compared to the control group (Fig. S2E). These observations provide evidence that miR-874 is a potent inhibitor of HCC through the DOR/EGFR/ERK pathway in vivo.

## Discussion

DOR is the predominantly expressed protein in HCC and has a pro-tumor effect. Our previous studies determined that downregulation of the DOR suppresses the proliferation, apoptosis, invasion, and migration of HCC cells and enhances sensitivity to 5-Fu. This suggests that the DOR could be a therapeutic target in HCC. Currently, the potential of miRNAs as anticancer therapeutic targets is being investigated extensively. However, there are no reports of miRNAs targeting the DOR.

In our study, we first demonstrated that miR-874 inhibits the DOR. We found that miR-874 expression was downregulated in HCC tissue and inversely correlated with DOR expression, tumor size, lymph node invasion, TNM stage, and metastasis. Furthermore, in vitro and in vivo experiments confirmed that miR-874 inhibits the proliferation and metastasis of HCC cells. We also found that miR-874 inhibits the DOR, which influences the EGFR–ERK pathway. The DOR–EGFR–ERK pathway is responsible for miR-874-triggered proliferation and migration inhibition.

miR-874 is located on chromosome 5q31.2, which is well known as a fragile site in the human genome that is often deleted in cancers^10^. miR-874 has been reported to be involved in cancer progression and development and functions as a tumor suppressor in several types of cancers, such as gastric cancer^11^, breast cancer^12^, colorectal cancer^13^, non-small-cell lung cancer^10^, and maxillary sinus squamous cell carcinoma^14^. However, the detailed biological function and underlying molecular mechanism of miR-874 in HCC has not been well explored. We found that miR-874 expression levels were downregulated in HCC tissues and cell lines, consistent with a recent report^14^, and that the restoration of miR-874 suppressed tumor growth and metastasis. This finding indicates that miR-874 may function as a tumor suppressor in HCC.

Among members of the opioid receptor superfamily, the DOR is most closely involved in the survival and proliferation of normal cells. Studies have shown that activation of the DOR can protect the liver from damage in cholestatic liver disease by promoting liver generation and liver cell proliferation^15,16^. In addition, the DOR is closely associated with cancer progression, and increased DOR expression has been detected in breast^5^, lung^4^, colorectal^17^, and liver cancer^6^.

Particularly in liver cancer, our previous study showed that upregulation of the DOR promotes liver cancer proliferation and metastasis both in vitro and in vivo^6^. Downregulation of the DOR enhances the sensitivity of drug-resistant HCC cells to 5‑FU^18^. We also found that activated DOR inhibits hydrogen peroxide-induced apoptosis in liver cancer cells^7^, suggesting that the DOR is involved in the proliferation, metastasis, and apoptosis of HCC. However, the epigenetic mechanism of DOR overexpression in HCC has rarely been reported. In the present study, we first discovered that miR-874 regulates the DOR. Overexpression of miR-874 using a mimic decreased DOR expression, resulting in suppressed cell proliferation and metastasis in HCC. A luciferase reporter assay indicated that the regulation of the DOR by miR-874 depended on its binding to the 3′-UTR of the DOR. Thus, the DOR was inhibited by miR-874, which had an onco-suppressive role in HCC cells.

We further studied the mechanism of miR-874/DOR inhibiting HCC progression. ERK was the first mitogen-activated protein kinase (MAPK) to be identified and is the most studied MAPK member. Studies have shown that the ERK signaling pathway is involved in HCC carcinogenesis^19^. Our previous studies demonstrated that DOR activation promotes ERK phosphorylation and inhibits human pancreatic cancer progression^20^. Moreover, the DOR may regulate apoptosis through activation of the ERK signaling pathway in HCC^7^. In the present study, the phosphorylation of ERK was downregulated by miR-874 overexpression, and ectopic DOR expression eliminated the promoting effect of miR-874 overexpression on phosphorylation level. This finding suggests that miR-874/DOR can inhibit ERK phosphorylation.

The EGFR is a receptor tyrosine kinase (RTK), and its overexpression occurs in the majority of HCCs, correlating with aggressive tumors, metastasis, and poor patient survival^21^. Fujioka reported that morphine-induced phosphorylation of the EGFR occurs via opioid receptors, leading to downstream ERK phosphorylation in lung cancer^4^. In our study, the EGF significantly restored the inhibition of phosphorylation of ERK induced by miR-101 overexpression, and the ERK inhibitor U1026 could not interfere with EGFR phosphorylation. This result suggests that miR-874/DOR may inhibit ERK phosphorylation via the EGFR. Moreover, we observed that the EGF required the repression of proliferation and migration induced by miR-874 overexpression, but U1026 can block this effect, suggesting that the EGFR–ERK pathway is responsible for miR-874-triggered proliferation and migration inhibition.

The exact mechanism by which miR-874/DOR inhibits EGFR phosphorylation is not clear in HCC. Fischer reported ligand-dependent EGFR signal transactivation by GPCRs in cancer cells^22^. The DOR is a type of GPCR, so it may activate EGFR phosphorylation though a GPCR-EGFR crosstalk mechanism, but this supposition requires further investigation.

On the basis of previous studies of the DOR, we identified miR-874 and determined its biological function in HCC in vitro and in vivo. We demonstrated that miR-874 promotes the proliferation and metastasis of HCC by targeting the DOR/EGFR/ERK pathway. This study not only complements the miRNA regulation study of HCC but also provides a new putative marker to monitor and treat HCC. Clearly, some problems remain to be resolved, for example, the direct cause of the downregulation of miR-874 in HCC and the exact mechanism by which miR-874/DOR inhibits EGFR phosphorylation. These issues may become the focus and direction of our future research.

## Material and Methods

### Patient samples

This study was reviewed and approved by the Ethical Committee of the Second Affiliated Hospital of Dalian Medical University. Written informed consent was obtained from all patients. The study included 120 patients with HCC who were 37 to 76 years old, all of whom underwent surgery from 2009 to 2012 at the Department of Hepatobiliary Surgery of the Hospital of Dalian Medical University. Clinicopathological characteristics were also examined, such as age, gender, tumor size, depth of invasion, tumor differentiation, lymph node invasion, TNM stage, and metastasis. Tumors were classified and graded based on the pTNM classification advocated by the International Union against Cancer.

### Cell lines and cell culture

The cell lines Bel-7402, SK-hep-1, HepG2, Huh7, Li-7, and LO2 were obtained from the American Type Culture Collection. All lines were cultured in modified Eagle’s minimal essential medium (Sigma-Aldrich Corp., China). All media were supplemented with 10% fetal bovine serum and 1% antibiotic/antimycotic solution (Biowest, France). All cell lines were cultured in 5% CO_2_ at 37 °C in incubators at 100% humidity.

### Immunohistochemistry

Tissues were fixed in formalin and embedded in paraffin for immunohistochemistry according to published protocols^23^. Briefly, the tissue sections were de-paraffinized in xylene and rehydrated using a graded ethanol series. To quench endogenous peroxidase activity, the sections were immersed in 0.3% peroxidase-methanol solution for 30 min. For antigen retrieval, the sections were pretreated with citrate buffer for 15 min at 100 °C in a microwave oven. The sections were hybridized with a primary antibody against DOR (Santa Cruz, USA) at 4 °C overnight at a dilution of 1:100. They were then visualized using the UltraVision Quanto Detection System HRP DAB kit (Thermo Scientific) according to the manufacturer’s protocols. The stained sections were counterstained with hematoxylin, and photomicrographs were captured using an Olympus BX51 microscope. To investigate the number of DOR-positive cells, we arbitrarily selected 10 high-power fields (×200) and counted the cancer cells under the microscope, as described previously^23^.

### ISH analysis

ISH analysis was performed according to a previously described method^24^. Antisense oligonucleotide probes for miR-874 (Exiqon Inc., Woburn, MA, USA) were used for ISH.

### Western blot

Tissues or cells were homogenized and lysed with lysis buffer (50 mM Tris–HCl, 137 mM NaCl, 10% glycerol, 100 mM sodium orthovanadate, 1 mM phenylmethylsulfonyl fluoride (PMSF), 10 mg/ml aprotinin, 10 mg/ml leupeptin, 1% Nonidet P-40, and 5 mM protease inhibitor cocktail; pH 7.4). After determining the protein concentration using a BCA assay, β-mercaptoethanol and bromophenol blue were added to the sample buffer for electrophoresis. The proteins were separated via 10% PAGE and transferred to polyvinylidene difluoride membranes (Bio-Rad, USA). The membranes were incubated in a primary antibody overnight at 4 °C. After incubation in a secondary antibody for 2 h, the reactive bands were visualized using an enhanced chemiluminescence system. The bands intensities were quantified using an image analysis system.

### Quantitative real time RT-PCR

Total RNA was extracted from the cells or tissues using TRIzol (Invitrogen, China) according to the manufacturer’s protocol. For mature miR-874 detection, total RNA was polyadenylated using poly(A) polymerase (Ambion, USA). Reverse transcription was performed using poly(A)-tailed total RNA, a reverse transcription primer, and ImPro-II Reverse Transcriptase (Promega, USA) according to the manufacturer’s instructions. The qRT-PCR was performed as described in the instructions provided with the Fast Start Universal SYBR Green Master Mix (Rox) (Roche Diagnostics GmbH Mannheim, Germany). The primers used for amplification were as follows: miR-874 forward (5′-GGCCCTGAGGAAGAACTGAG-3′), reverse (5′-TGAG ATCCAACAGGCCTTGAC-3′), and DOR forward (5′-ACCAAGATCTGCGTGTTCCT-3′), reverse (5′-CGATG ACGAAGATGTGGATG-3′). U6 or β-actin was used as the internal control, and other specific primers were purchased from Invitrogen.

### Cell-cycle analysis

Cells were harvested at 48 h after transfection and then washed with phosphate-buffered saline solution (PBS) and fixed in 70% ethanol at 4 ℃ overnight. After fixation, the cells were washed twice with PBS before incubation in propidium iodide/RNase A solution (5 μg/ml propidium iodide and 100 mg/ml RNase A) at room temperature in the dark for 30 min. The stained cells were analyzed using a FACSCalibur flow cytometer (Becton-Dickinson, USA), and the analysis was completed within 30 min.

### MTT assay

At 24 h after transfection, cells were seeded on a 96-well plate at a density of 1 × 10^3^ cells per well. After incubation for 1, 2, 3, 4, 5, and 6 days at 37 ℃ in a humidified incubator, 20 μl of MTT (5 mg/ml in PBS) was added to each well, and the cells were incubated for another 4 h. After removing the medium, 150 μl of DMSO was added to each well. The absorbance at a wavelength of 540 nm was recorded using a microplate reader.

### miRNA and siRNA transfection

The miRNA-874 mimic and negative control were obtained from GenePharma (Shanghai, China). The sequence of the miR-874 mimic was 5′-CUGCCCUGGCCCGAGGGACCGA-3′. Cells (5 × 10^5^ cells/2 ml/well) were seeded at 60% confluence in a six-well plate. After 48 h, the miRNA-874 mimic or negative control was transfected into cells using Lipofectamine 2000 (Invitrogen, USA) at a final concentration of 50 nM according to the manufacturer’s instructions. The siRNA targeting the DOR, with a sequence of 5′-AAGACTCTGAATGCAGTTGCT-3′, and the miR-874 inhibitor were purchased from GenePharma. The transfection of siRNA and the miR-874 inhibitor was performed as described above. The final siRNA and miR-874 inhibitor concentrations were 100 and 50 nM, respectively.

### Vector constructs, transfections, and assays

A DOR expression plasmid (pcDNA3.1-DOR) containing the coding sequence but lacking the 3′-UTR was constructed using PCR-generated fragments and the pcDNA3.1 (+) vector. The 3′-UTR of DOR mRNA and a mutant variant were generated through q-PCR and cloned into the *Xba*I site of a pGL3-basic vector (Promega) and were designated DOR-wt-3′-UTR and DOR-mt-3′-UTR, respectively.

For the luciferase activity assay, SK-hep-1 cells were seeded in 6-well plates and transfected with the pGL3 reporter vector (250 ng/well), pRL-TK luciferase reporters (25 ng/well), and miR-874 mimic (100 ng/well) or a negative control vector using Lipofectamine 2000 (Invitrogen, Carlsbad, CA, USA) 24 h later. Luciferase activity levels were measured using a dual-luciferase reporter assay kit (Promega) according to the manufacturer’s instructions. For rescue assays, SK-hep-1 cells expressing miR-874 were transfected with pcDNA3.1-DOR, and the cells were collected to perform in vitro assays.

### Cell migration and invasion assays

Cells were seeded on six-well plates, incubated in their respective complete culture medium, and grown to confluence overnight. The cells were scratched using a standard 200 μl tip, and the debris was removed by washing the cells with serum-free medium. Serial photographs were obtained at different time points using a phase contrast microscope. The cell invasion assay was performed using Matrigel-coated transwell chambers (8-μm pore size, BD Biosciences, USA). Cancer cells were seeded above the Matrigel matrix in the upper chamber, and the bottom chamber was filled with culture medium containing a chemoattractant. The cells that permeated the Matrigel-coated membrane after 24 h were fixed with paraformaldehyde and then stained with crystal violet.

### Colony formation assays

The cells were seeded in 6-well plates at 5 × 10^2^ per well and incubated for 2 weeks for the colony formation assay. The cells were washed twice with PBS, fixed with methanol/acetic acid (3:1, v/v), and stained with 0.5% crystal violet (Sigma, China). The number of colonies was counted under a microscope (Olympus IX81, Japan).

### Lentivirus transduction

Lentiviral pEZX-MR04 plasmids expressing miR-874 or negative control miRNA were purchased from GeneCopoeia. Lentivirus expressing miR-874 or negative control miRNA was co-transfected into HCC cells using EndoFectin Lenti transfection reagent according to the manufacturer’s instructions. After culturing for 48 h, the lentiviral particles in the supernatant were harvested and filtered via centrifugation at 500 × *g* for 10 min. HCC cells were transduced with lentivirus expressing miR-874 or negative control miRNA. To select stably transduced cells, the cells were resuspended and cultured in the presence of puromycin (2 μg/ml) for 2 weeks, and qRT-PCR was performed to determine the level of miR-874 expression.

### In vivo tumor growth assay

All animal experiments complied with the policy of Dalian Medical University on the care and use of laboratory animals. Six-week-old male BALB/c nude mice were obtained (Shanghai Slac Laboratory Animal Co. Ltd., China) and bred under specific pathogen-free conditions. SK-hep-1 cells stably overexpressing miR-874 or empty vector were subcutaneously injected into the flank region of the mice (6 mice per group). Over a period of 5 weeks, tumor formation in the mice was observed by measuring the tumor volume. The tumors were excised and weighed.

### Statistical analysis

All values are expressed as mean ± standard deviation (SD). The significance of the differences was determined by one-way ANOVA or Student’s *t*-test. The *χ*^2^ test was used to evaluate the relationship between expression and clinicopathological characteristics. Spearman’s correlation coefficient was used to calculate correlations between two groups. Kaplan–Meier analysis was employed for survival analysis, and the differences in survival probabilities were estimated using the log-rank test. *p* < 0.05 was considered significant. Statistical analyses were performed using SPSS version 17.0 (SPSS, Inc., USA).

## Electronic supplementary material

Figure S1

Figure S2

Supplementary Figure Legends
