# Supplementary figures and images for: microRNA-874 suppresses tumor proliferation and metastasis in hepatocellular carcinoma by targeting the DOR/EGFR/ERK pathway
Source: Cell Death Dis. 2018 Jan 26;9(2):130. doi: 10.1038/s41419-017-0131-3 (PMC5833540; doi:10.1038/s41419-017-0131-3)

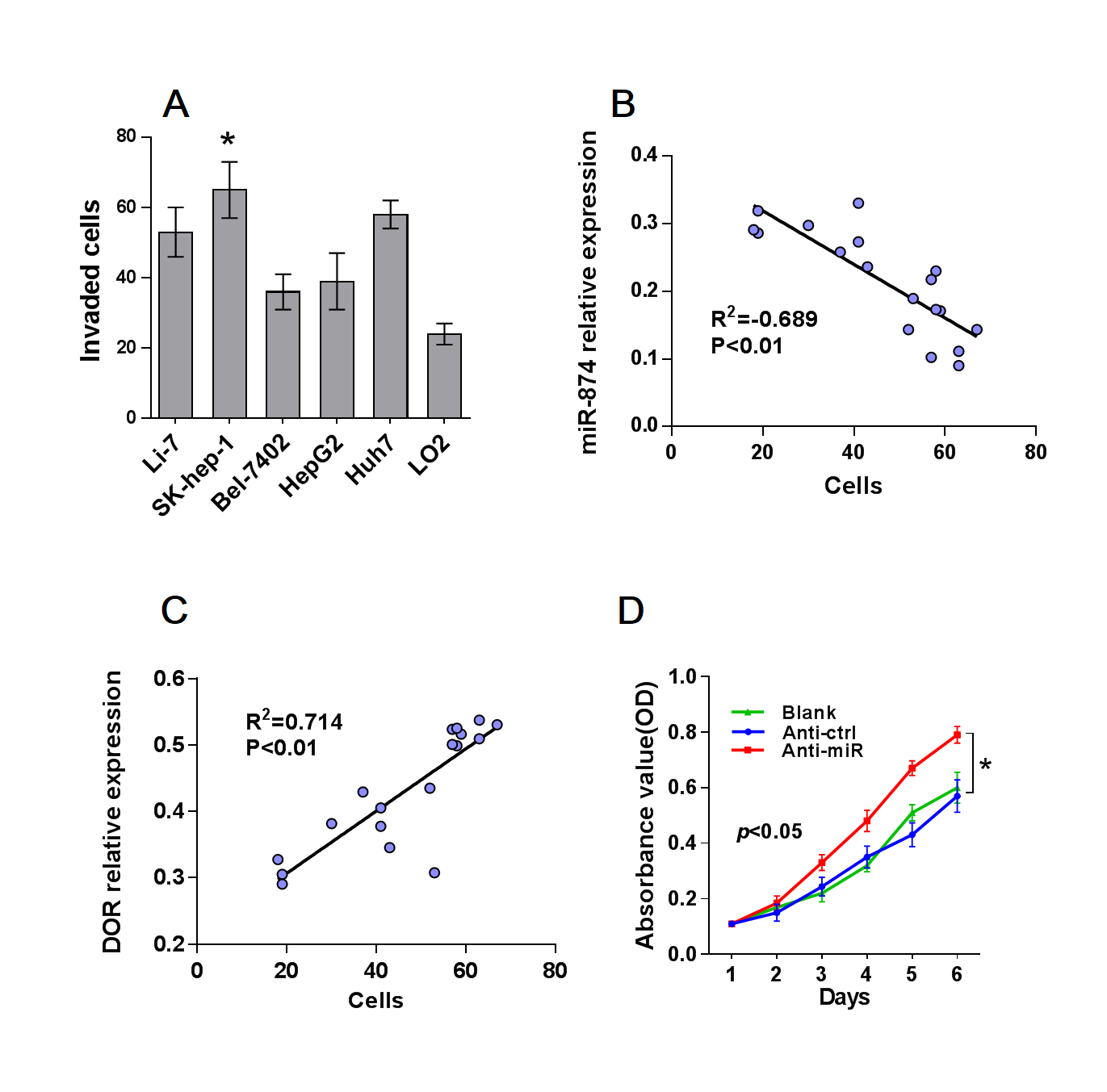

Supplement: Supplementary file 1 — Figure S1 [file 41419_2017_131_MOESM1_ESM.tif]

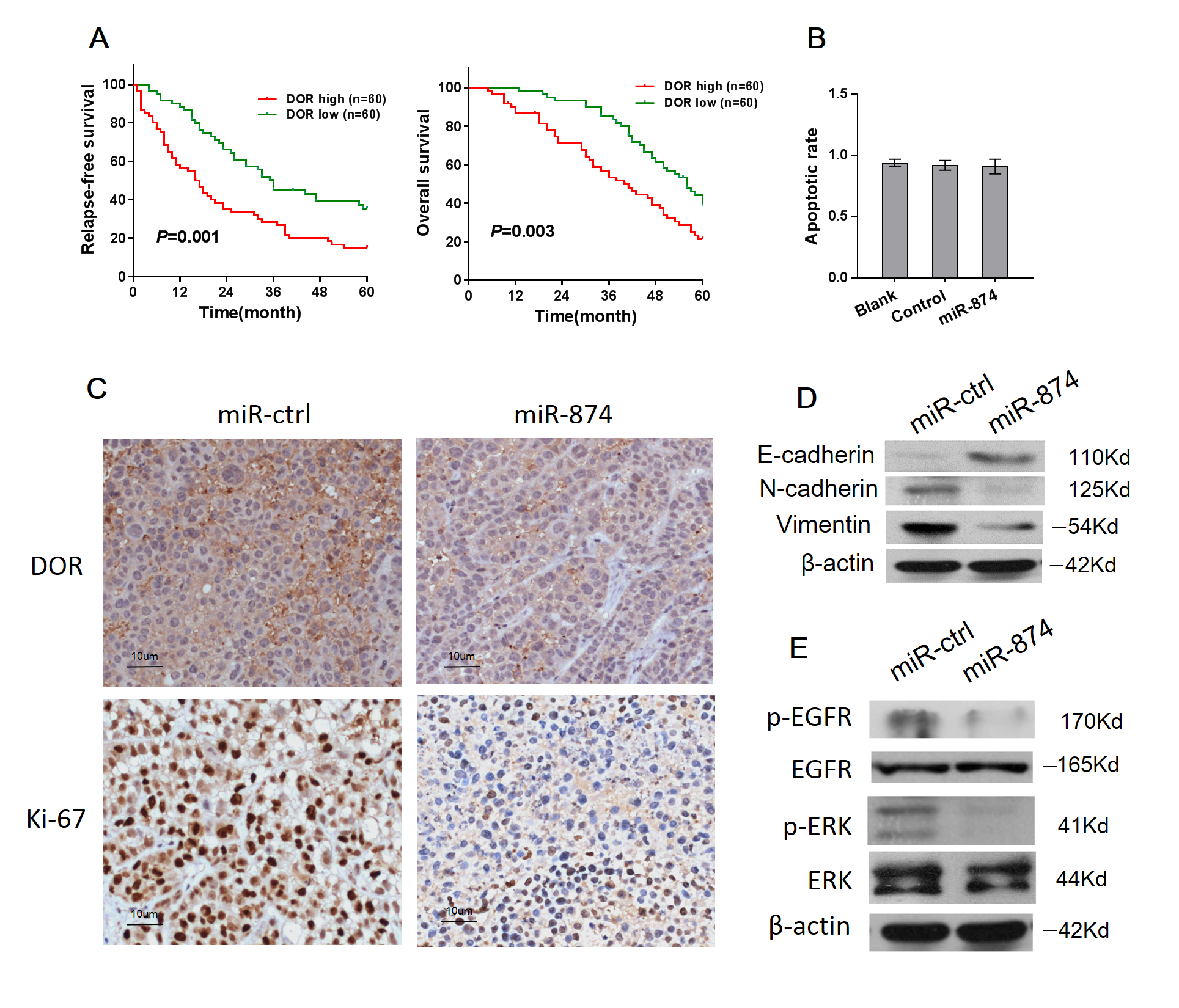

Supplement: Supplementary file 2 — Figure S2 [file 41419_2017_131_MOESM2_ESM.tif]
