# Supplementary material for: microRNA-874 suppresses tumor proliferation and metastasis in hepatocellular carcinoma by targeting the DOR/EGFR/ERK pathway
Source: Cell Death Dis. 2018 Jan 26;9(2):130. doi: 10.1038/s41419-017-0131-3 (PMC5833540; doi:10.1038/s41419-017-0131-3)
Supplement: Supplementary file 3 — Supplementary Figure Legends [file 41419_2017_131_MOESM3_ESM.docx]

**Supplementary Figure Legends**

**Figure S1.** (A) The invasion of five HCC cell lines was examined via transwell assay. SK-hep-1 cells exhibited significantly higher invasive capacity than the other cell types. Normal liver LO2 cells were used as a control. (B, C) The level of miR-874 was negatively correlated with invasiveness of colon cancer cells (r=-0.689), whereas mRNA levels of the DOR were positively correlated with invasiveness of colon cancer cells (r=0.714). (D) miR-874 knockdown with the miR-874 inhibitor promoted proliferation of SK-hep-1 cells. Data are shown as the mean ± S.D (*****, *p*<0.05).

**Figure S2.** (A) Kaplan-Meier analysis revealed that OS and RFS rates of the low DOR expression group were lower than those of the high miR-874 expression group (B) Apoptotic assay showed that miR-874 overexpression has few effects on cell apoptosis. (C) Analysis of DOR and Ki-67 expression in resected tumors of the miR-874 and control groups. (D) Western blot showed that miR-874 overexpression increased expression of E-cadherin and decreased expression of N-cadherin and vimentin in SK-Hep-1 cells. (E) Phosphorylation of the EGFR and ERK were down-regulated in resected tumors of the miR-874 group, compared to the control group. Data are shown as the mean ± S.D (*****, *p*<0.05).
